# High-Resolution 3T MR Imaging of the Triangular Fibrocartilage Complex

**DOI:** 10.2463/mrms.rev.2016-0011

**Published:** 2016-08-16

**Authors:** Donald von Borstel, Michael Wang, Kirstin Small, Taiki Nozaki, Hiroshi Yoshioka

**Affiliations:** 1 Department of Radiological Sciences, University of California, Irvine, UCI Medical Center 101 The City Dr. South, Route 140, Orange, CA 92868, USA; 2 Department of Radiology, Brigham and Women’s Hospital, Boston, MA, USA; 3 Department of Radiology, St. Luke’s International Hospital, Tokyo, Japan

**Keywords:** triangular fibrocartilage complex, wrist MRI, high-resolution MRI, isotropic 3D imaging

## Abstract

This study is intended as a review of 3Tesla (T) magnetic resonance (MR) imaging of the triangular fibrocartilage complex (TFCC). The recent advances in MR imaging, which includes high field strength magnets, multi-channel coils, and isotropic 3-dimensional (3D) sequences have enabled the visualization of precise TFCC anatomy with high spatial and contrast resolution. In addition to the routine wrist protocol, there are specific techniques used to optimize 3T imaging of the wrist; including driven equilibrium sequence (DRIVE), parallel imaging, and 3D imaging. The coil choice for 3T imaging of the wrist depends on a number of variables, and the proper coil design selection is critical for high-resolution wrist imaging with high signal and contrast-to-noise ratio. The TFCC is a complex structure and is composed of the articular disc (disc proper), the triangular ligament, the dorsal and volar radioulnar ligaments, the meniscus homologue, the ulnar collateral ligament (UCL), the extensor carpi ulnaris (ECU) tendon sheath, and the ulnolunate and ulnotriquetral ligaments. The Palmer classification categorizes TFCC lesions as traumatic (type 1) or degenerative (type 2). In this review article, we present clinical high-resolution MR images of normal TFCC anatomy and TFCC injuries with this classification system.

## Introduction

Magnetic resonance (MR) imaging has taken on the premier role in the diagnosis of internal derangement of joints. However, the diagnosis of injuries to the ligamentous structures of the wrist, especially the triangular fibrocartilage complex (TFCC), can be a challenge because it is a small and complex structure. The recent advances in MR imaging, including high field strength magnets, multi-channel coils, and isotropic 3-dimensional (3D) sequences have enabled the visualization of precise TFCC anatomy with high spatial and contrast resolution. In this article, we review recent routine and arthrogram MR protocols of the wrist including the isotropic 3D sequence. Then, normal TFCC anatomy and traumatic/degenerative lesions of the TFCC are described along with high-resolution 3T MR images.

## Protocols for 3T General Imaging of the Wrist

To achieve optimal imaging of the detailed anatomy of the wrist, especially the TFCC structures, a balance of maximum spatial resolution, signal-to-noise (SNR), and contrast resolution must be achieved. These variables are related and all influenced by changes in specific parameters such as field of view (FOV), matrix size, slice thickness, bandwidth, echo time, repetition time (TR), applied magnetic field strength, and pulse sequence selection.^1^ The applied magnetic field strength in this study is presumed to be 3Tesla (T). There are higher field systems (7T) being evaluated in the research realm.^2^ However, 3T imaging has become more widely used and is a practical scanner for the detailed imaging of the wrist.

### Non-contrast MR imaging of the wrist

To evaluate the minute, detailed anatomy that comprises the TFCC, high spatial resolution sequences are necessary. Using 3T imaging increases the SNR by two times that of a 1.5T magnet.^3,4^ There is a linear relationship between the applied field strength and signal. Using 3T field strength allows for increased spatial resolution and a decreased scan time. Decreasing the overall scan time presumably yields improved image quality secondary to less motion artifacts.

Specific alterations can be made to increase the overall spatial resolution. Specifically, increasing the matrix size for a given FOV or decreasing the FOV for a fixed matrix improves overall spatial resolution. These changes, however, can result in decreased SNR and degradation of imaging quality.^1^ This decrease in SNR can be negated when using 3T imaging or specialized coils, which will be discussed later in this study.

The basic routine sequence protocols for 3T imaging of the wrist include proton density (PD)-weighted imaging, T_2_-weighted imaging, T_2_^*^ gradient-recalled echo sequences, and other fluid-sensitive sequences with fat suppression. Most commonly, the sequences are acquired with conventional 2D technique. However, 3D imaging techniques specific to the wrist and hand have recently become more common. The authors’ routine wrist protocol includes coronal PD and fat suppressed (FS) PD, axial PD and FS PD or FS T_2_, sagittal PD and FS PD, and coronal isotropic 3D FS PD imaging. A detailed overview of this protocol is shown in Table 1.

### MR arthrogram of the wrist

MR arthrogram (MRA) of the wrist is generally preferred over conventional MR imaging or computed tomography arthrography (CTA).^5,6^ This is because of its excellent intrinsic contrast resolution, ability to properly diagnose early stage carpal instability, and to differentiate TFCC ligament tear types for the determination of prognosis and possible treatment.^7^ Also, CTA does not evaluate marrow abnormalities or extra-articular soft tissue abnormalities. Intra-articular contrast agents allow better evaluation of the TFCC and interosseous ligaments of the wrist.^5^ The overall accuracy of detecting the intrinsic wrist ligament and TFCC tears has been shown to be statistically enhanced using MRA.^6^ Also, it has been shown that the sensitivity, specificity, and accuracy of MRA using a 3T scanner have been higher than most studies performed at 1.5T, suggesting that higher field MR imaging can improve the detection of intrinsic ligament and TFCC tears.^8^

There are disadvantages to MRA in comparison with standard MR imaging, including increased cost and the invasive nature of contrast injection into a joint. This procedure can be a time-consuming procedure for the radiologist and any joint injection involves inherent small risk factors for infection, synovitis, and increased radiation exposure. These minor disadvantages are often outweighed by the significantly improved diagnostic accuracy of MRA, particularly in cases where detailed evaluation of cartilage or ligaments is vital.

The typical sequence protocol that our department uses for the routine wrist MRA is shown in Table 2.

## Specific Techniques for 3T Imaging of the Wrist

**Fig 1.**
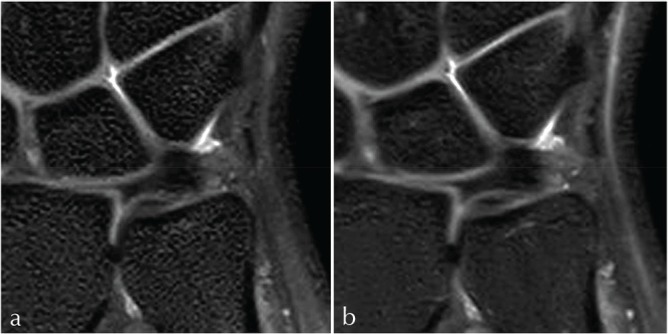
(**a, b**) 3-dimensional (3D) isotropic coronal sequences of the triangular fibrocartilage complex (TFCC) utilizing the SENSE parallel imaging and driven-equilibrium sequence (DRIVE) technique to achieve a high signal-to-noise (SNR) with maximum spatial and contrast resolution. This is done with a relatively short repetition time (TR) achieving a clinically useful scan time while obtaining approximately 150 coronal images of the wrist. The images were obtained with (**a**): TR = 1250, TE = 38, ETL = 70, voxel size = 0.35, FOV = 7 cm, scan time = 5 min 16 s and (**b**): TR = 1400, TE = 29, ETL = 70, voxel = 0.35, FOV = 7 cm, and scan time = 4 min 19 s.

There are specific techniques used to optimize 3T imaging of the wrist, which are increasingly used throughout the realm of clinical imaging. These techniques allow for 3T imaging of the wrist to attain maximum spatial and contrast resolution while optimizing SNR within a clinically practical scan time. These include the driven equilibrium sequence (DRIVE), parallel imaging, and 3D imaging (Fig. 1)

### DRIVE sequence

Driven equilibrium is a novel imaging sequence that makes it possible to reduce scan time and improve image quality on T_2_-weighted imaging. In rapid imaging, substances with long T_1_ and T_2_ values (i.e. joint effusion, cerebrospinal fluid, urine, or bile) do not fully recover their longitudinal magnetization by the end of every TR interval. To obtain high fluid signal and consistent contrast after each excitation, the TR must be long enough to restore the fluid magnetization. Therefore, long TR is needed to maintain the high signal of fluids in these sequences.

**Fig 2.**
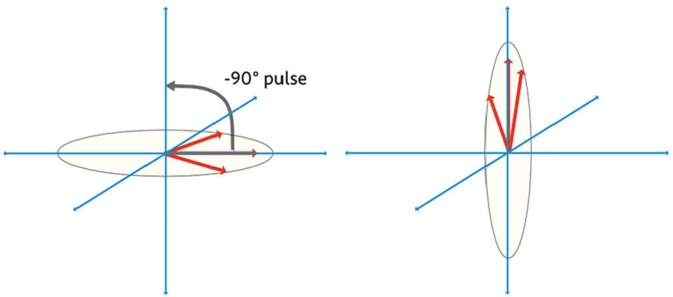
The driven equilibrium technique applies a −90° radiofrequency (RF) pulse which accelerates T_1_ recovery.

After a number of cycles, the saturation begins to occur and the normal high T_2_ signal of these fluids is decreased, even when employing long TR. Driven equilibrium techniques are useful in restoring this signal.^9^ A −90° radiofrequency (RF) pulse is applied, in combination with a gradient refocusing pulse and a spoiling gradient, to help restore longitudinal magnetization at the end of an MR sequence. The −90° pulse tips the residual transverse magnetization back into the z-direction (vertical axis). After this −90° pulse, a short amount of time is needed until T_1_ relaxation of the fluid and the other tissues is complete, and a subsequent excitation pulse can be applied. This essentially “jump starts” the T_1_ relaxation for the next TR interval and accentuates signal from fluids. Thus, the TR is shortened by a factor of three to four. This sequence is widely employed in other areas outside of joint imaging, including spinal imaging, MR urography, and MR cholangiopancreatography (MRCP) (Fig. 2).^10–12^

Driven equilibrium pulses may be used in conjunction with virtually any imaging sequence, but most commonly is utilized with fast spin echo (FSE) imaging. Major MR vendors offer these sequences under differing trade names: Siemens’ RESTORE (Siemens Healthineers, Erlangen, Germany), GE’s Fast Recovery Fast Spin Echo (FRFSE, General Electric Healthcare, Waukesha, WI, USA), Phillips’ DRIVE (Philips Healthcare, Best, The Netherlands), Hitachi’s Driven equilibrium FSE (Hitachi Medical Corporation, Tokyo, Japan), and Toshiba’s T_2_ plus FSE (Toshiba Medical Systems Corporation, Tochigi, Japan). Also, the generic acronym driven equilibrium Fourier transform (DEFT) has been used in literature.

### Parallel imaging

Parallel imaging is a uniquely designed technique using spatial data that are derived from phased array coil elements to construct a portion of k-space. Using the coil elements to obtain k-space data diminishes the burden of filling lines of k-space data with individually acquired phase gradients. The end result of the data acquisition technique is a decrease in scan time and a decrease in the specific absorption rate (SAR) because of the reduced number of RF pulses required for transmission to the patient.

**Fig 3.**
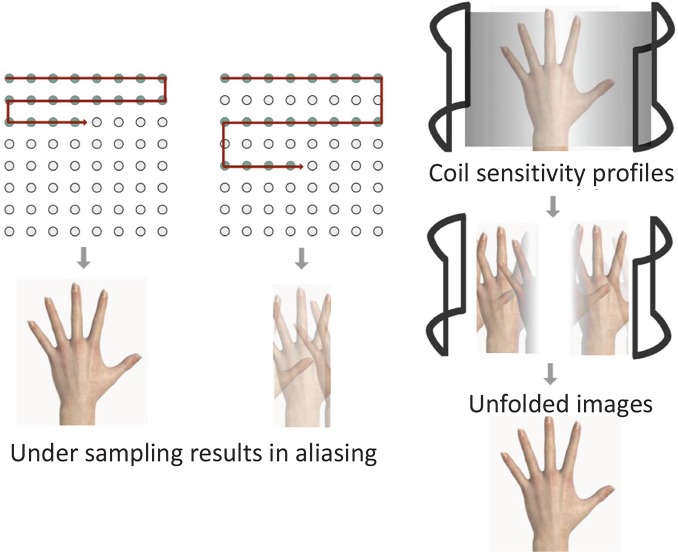
Parallel imaging involves under sampling of k-space which results in aliasing. The individual coil sensitivity profiles are used to generate the final image.

There are currently two basic techniques of k-space parallel imaging which have been developed and further modified as major manufacturers of MR imaging systems use these versions. The first is k-space parallel imaging before Fourier transformation which includes simultaneous acquisition of spatial harmonics (SMASH), generalized autocalibrating partial parallel acquisition (GRAPPA), and autocalibrating reconstruction for the Cartesian imaging (ARC). The other technique is image domain parallel imaging after Fourier transformation which includes sensitivity encoding (SENSE), mSENSE, and array coil spatial sensitivity encoding (ASSET). These groups of different approaches to parallel imaging yield similar benefits.^13^ For example, SMASH imaging is performed by only filling a fraction of the lines of k-space with the phase-encoding gradient, whereas the remaining unfilled k-space data is filled by individual coil elements. The final k-space construct is used to create an image. The SENSE imaging conversely uses each coil element to obtain an aliased image by under sampling k-space. These aliased images are then combined with the k-space acquired with phase-encoding gradients. All of the aliased images are combined mathematically to create a final image (Fig. 3).^3^ The vendors have combined variations of these two techniques for multiple modified versions of parallel imaging.

Parallel imaging can be applied in varying degrees to reduce scan time by factors of two, four, and more depending on the capabilities of the MR imaging system and the number of coil and receiver channels. This degree of time reduction has been called the acceleration factor.^3^ The net reduction in the amount of time required to obtain the MR image is related to the number of independent coil channels within the array. Multiple channels are required to process the data independently, and in principle, an 8-channel coil would be able to image eight times as fast, assuming an ideal geometry. However, practical considerations limit the image acceleration to values below the theoretical maximum.^14^ The author’s department uses an acceleration factor of two with an eight-channel phased array coil.

The disadvantage of using parallel imaging is a decrease in overall SNR that is proportional to the acceleration factor. As the acceleration factor increases with parallel imaging, the SNR decreases ( $\text{SNR}/\sqrt{\text{accelertion}\;\text{factor}}$ ). For example, by using an acceleration factor of four, the overall SNR would decrease by 50%.^3^ Consequently, a 3T magnet is the ideal combination for parallel imaging because of the increased signal which compensates for the expected decrease in SNR.^15^ In the future, it can be assumed that more phased array coils will be available with an increased number of channels per coil.

### 3D imaging

Conventional imaging of the musculoskeletal system has been historically performed with 2D multi-slice acquisitions. FSE sequences are a commonly used sequence because of their excellent visualization of anatomy and pathology.^16,17^ The drawback of 2D FSE imaging is the voxels obtained are anisotropic; resulting in thick slices in comparison with the in-plane resolution. This ultimately leads to volume artifact. Also, the acquisition of anisotropic voxels does not allow the opportunity for reformatting into various planes.

These limitations of 2D FSE imaging can be overcome by isotropic 3D MR imaging, which allows precise and detailed assessment for small joint evaluation.^18^ The cross-sectional images may be reformatted into an arbitrary plane without substantial degradation of image quality (see Anatomy and Traumatic and Degenerative Lesions of the TFCC below). This allows for a more complete assessment of the wrist ligaments and small structures of the TFCC. This also permits cross-referencing of very small structures between multiple planes without misregistration. The thin contiguous sections with isotropic 3D MR imaging eliminate the partial volume averaging which occurs with 2D FSE imaging.^19^ And finally, 3D MR imaging allows a decreased acquisition time as a function of obtaining a single isotropic 3D sequence versus three (axial, coronal, and sagittal) 2D sequences.^20^ Yamabe et al.^21^ found that it took approximately 23 min to obtain imaging using a 2D sequence in three planes with and without fat suppression, while taking just 10 min to obtain equivalent isotropic 3D FSE images.

Pitfalls of isotropic 3D MR imaging include imaging blur and slight degradation after reformatting.^1^ However, even though isotropic 3D images have shown more image blur than comparable 2D MR images with slight imaging degradation after reformatting to sagittal and axial planes, this has not shown to affect delineation of small structures of the wrist, including the intrinsic ligaments and TFCC. The isotropic 3D MR imaging of the wrist is clinically feasible and useful within a reasonable scan time.^21^

There are two techniques which are generally used for 3D imaging; the FSE imaging and gradient echo (GE) imaging. GE type isotropic 3D MR imaging can be performed with a shorter acquisition time. However, GE 3D imaging does not provide a satisfactory contrast, especially between bone marrow edema and soft-tissue edema.^22^ FSE images present better imaging contrast to diagnose TFCC injury. The author’s institution uses isotropic 3D FSE MR imaging. However, FSE isotropic 3D MR imaging requires longer scan time than GE isotropic 3D MR imaging. Therefore, the techniques for reducing scan time have to be combined to accomplish a clinically useful scan time. These techniques include longer echo train length (ETL), DRIVE technique, and parallel imaging as mentioned above.

## Coils Used and Patient Positioning

There are currently a variety of commercially available coil designs dedicated for high-resolution imaging of the wrist. Proper coil design selection is critical for high-resolution wrist imaging with optimized SNR and contrast-to-noise ratio (CNR). The coil choice for 3T imaging of the wrist depends on the desired number of channels and elements, the size of the structure to be imaged, the manufacturer and scanner type, and the physical design of the coil for intended patient positioning. In general, the SNR is optimized by selecting a coil size that is ≈1.5 times the size of the anatomic region to be imaged. The benefit of selecting a coil which has multiple elements and a multichannel design is the increased SNR of a smaller coil, but with the increased FOV a larger coil permits. Phased array coils are a specially designed coil that uses a series of highly specialized coil elements to obtain signal individually. These coil elements have the potential to provide spatial data for k-space construction.^3^

**Fig 4.**
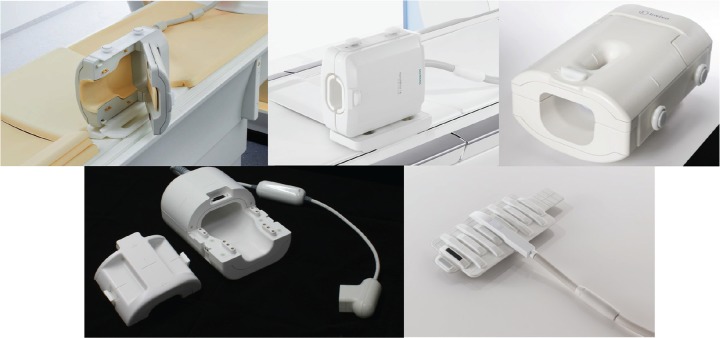
(left to right, top to bottom) Philips 8 channel coil (Philips Healthcare, Best, The Netherlands), Siemens 16 channel hand/wrist coil (Siemens Healthineers, Erlangen, Germany), GE 8 channel coil (General Electric Healthcare, Waukesha, WI, USA), Hitachi 10 channel coil (Hitachi Medical Corporation, Tokyo, Japan), Toshiba 16 channel flexible coil (Toshiba Medical Systems Corporation, Tochigi, Japan).

Multiple coils from different manufacturers are shown in Fig. 4. The physical design of the coil housing structure can limit patient positioning and indirectly determine if supine or prone positioning, or neutral or pronation wrist positioning is to be used. This change in positioning has associated variation in the spatial relationship of imaged structures, specifically, the TFCC and the extensor carpi ulnaris (ECU) tendon. The specific coil design can be optimized for wrist imaging with the patient in the so-called “superman” position with the pronation forearm prone positioning. This allows the wrist to lie within the isocenter of the magnet. This permits for improved uniform fat saturation and a higher SNR than when the wrist is centered outside the magnet isocenter. However, this position is difficult to maintain for long periods of time and the patient will be more uncomfortable, leading to the increased motion artifact. The image quality with a surface coil used in supine positioning has been shown to be superior, quantitatively and qualitatively, when compared to the prone position with a wrist volume coil in the magnet isocenter.^23^ This discomfort of the patient and resulting motion-degraded images is why the authors’ department performs the wrist imaging in the supine position with the wrist in neutral position. Many recent coils are optimized for imaging the patients lying in a supine position with their wrist by their side in neutral position.^1^

Especially for imaging of the TFCC, the microscopy coils can provide extremely high spatial resolution wrist imaging. The limitation of these coils is their inherently small FOV, which may only partially image the region of interest depending on usage. An option to compensate for this limitation is by using a microscopy coil in combination with a larger coil, such as a flexible coil. This allows imaging of both the hand and detailed imaging of the wrist, although with variation in diagnostic accuracy and spatial resolution.^1^

## Anatomy

In the 19th century, the existence of the triangular fibrocartilage (TFC) of the wrist was described by Weitbrecht and Henle as a “fibrocartilaginous structure, interposed between the inferior radio-ulnar and radio-carpal joint, triangular-shaped, extending from the medial edge of the lower end of the radius and being attached to the ulnar styloid process.” This description has been generally accepted up to the present time, and adopted by most anatomy textbooks.^24^ The clinical interest in the TFC was first generated by Lippmann’s report stating that one of the primary causes of the wrist instability following a Colles’ fracture is an injury to the TFC.^25^ Then, Palmer et al. described the “triangular fibrocartilage complex (TFCC)” of the wrist, composed of the articular disc (disc proper), the dorsal and volar radioulnar ligaments, the meniscus homologue, the ulnar collateral ligament (UCL), the sheath of the ECU, the ulnolunate ligament, and the ulnotriquetral ligament.^26^ The TFCC has been shown to have three major functions.^27^ First, it is the major stabilizer of the distal radioulnar joint. Second, it is an ulnar stabilizer of the radio-ulno-carpal joint. Third, it functions as a cushion for the ulnar carpus, carrying the axial load of the forearm. The “TFCC” is an appropriate term when considering these differing functions.

### Triangular fibrocartilage

**Fig 5.**
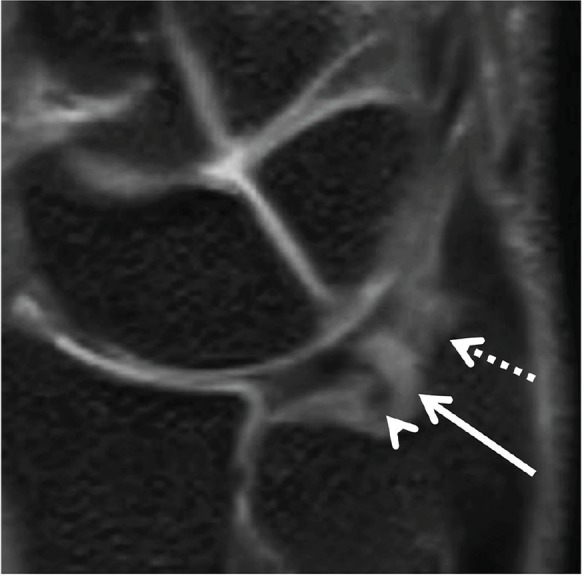
The ulnar attachment of the triangular fibrocartilage (TFC) (triangular ligament) on the oblique coronal 3-dimensional (3D) multiplanar reconstruction (MPR) image. The distal lamina inserts to the tip of the ulnar styloid process (dotted arrow). The proximal lamina inserts to the fovea of the ulnar styloid process (arrowhead). The region of high signal intensity between the two lamina shows the ligamentum subcruentum (arrow).

**Fig 6.**
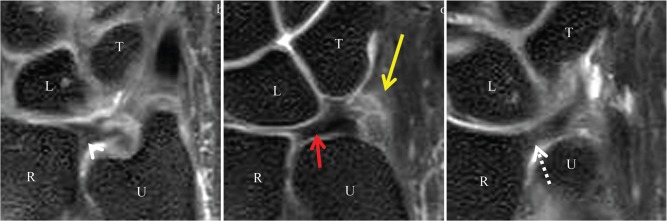
Triangular fibrocartilage (TFC) and radioulnar ligaments on isotropic 3-dimensional (3D) coronal fat suppressed (FS) proton density (PD)-weighted images. (**a**) The dorsal radioulnar ligament (arrowhead), (**b**) The central disc (red arrow), and (**c**) The volar radioulnar ligament (dotted arrow). The ulnomeniscus homologue (yellow arrow) merges from the tip of the ulnar styloid process, and inserts to the ulnar side of the triquetrum and lunate. L, lunate; T, triquetrum; R, radius; U, ulna.

The TFC is the largest component of the TFCC. It is composed of the fibrocartilage disc and the dorsal and volar radioulnar ligaments and has an asymmetric rectangular shape. In other words, the central portion is thin and the peripheral portion is thick, like a biconcave lens. The TFC arises from the sigmoid notch of the radius and inserts into the ulnar styloid process, extending along its entire surface to the most proximal fovea of the styloid process. At the ulnar attachment of the TFC, it usually bifurcates into a proximal and distal lamina that can be visualized on coronal or oblique coronal MR imaging (Fig. 5). It is difficult to macroscopically delineate the two separate laminae in cadaveric specimens. The “triangular ligament” is a synonym of the ulnar side of the TFC.^28–30^ The origin of the term “triangular ligament” derives from the ulnar attachment convergence of the two laminae from the articular disc. There is fibrovascular connective tissue named the “ligamentum subcruentum,” which intervenes between these two laminae (Fig. 5).^31^ There is a normally hyperintense signal within the ligamentum subcruentum on fluid-sensitive sequences, because of its rich vascularity.^30,32^ The TFC resembles the meniscus of the knee given its vascular distribution and tissue components. That is, the periphery of the disc is relatively vascular, and there is a predominantly avascular central portion of the disc. Because the central portion of the disc is not only avascular, but also very thin, it tends to perforate with age-related degeneration. The TFC is mainly assessed on coronal or oblique coronal MR images (Figs. 5, 6).

### Radioulnar ligament

The peripheral portion of the disc proper is thickened and it has longitudinally oriented collagen fibers. These fibers travel from the volar and dorsal aspects of the sigmoid notch of the radius to the base of the ulnar styloid process.^27^ These thickened fibrous bands are described as the dorsal and volar (palmar) distal radioulnar ligaments. They are mainly assessed on high-resolution coronal or axial MR images (Fig. 6).

### Extensor carpi ulnaris tendon

**Fig 7.**
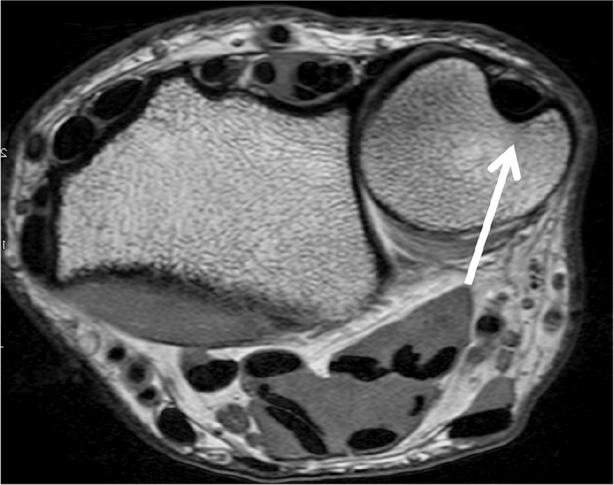
The extensor carpi ulnaris (ECU) in the neutral position on axial proton density (PD)-weighted image. The ECU (arrow) locates dorsal side of the ulnar styloid.

The ECU originates from the lateral epicondyle of the humerus and the posterior border of the ulna. It inserts at the base of the fifth metacarpal. The ECU subsheath is an important stabilizer of the ulnar side of the TFCC, and it changes in position between supination and pronation.^33^ In neutral position, it travels dorsally and medially to the ulnar styloid process, close to the UCL (Fig. 7).

### Ulnomeniscal homologue

The ulnomeniscal homologue is a fibrous tissue on the ulnar aspect of the TFCC, which could be subdivided into styloid, radioulnar, and collateral components.^34^ It was first described in 1970 by Lewis et al.^35^ The ulnomeniscal homologue merges from the tip of the ulnar styloid process and inserts to the ulnar aspect of the triquetrum and lunate.

From the viewpoint of comparative anatomy, the ulnomeniscal homologue does not exist with primates. The ulna styloid process is a part of the wrist joint in primates, directly articulating with the triquetrum and pisiform. With evolution, the ulna withdraws, and the ulnomeniscus homologue merges between the ulnar styloid process and carpal bones. As a result, humans have acquired the motions of supination and pronation.^35^

The ulnomeniscal homologue can be assessed on high-resolution coronal MR imaging, and is best visualized with MRA with the wrist in neutral position or radial deviation. The visualization of the ulnomeniscal homologue is highly dependent on the wrist positioning during imaging (Fig. 6).^34^

### Ulnar collateral ligament

**Fig 8.**
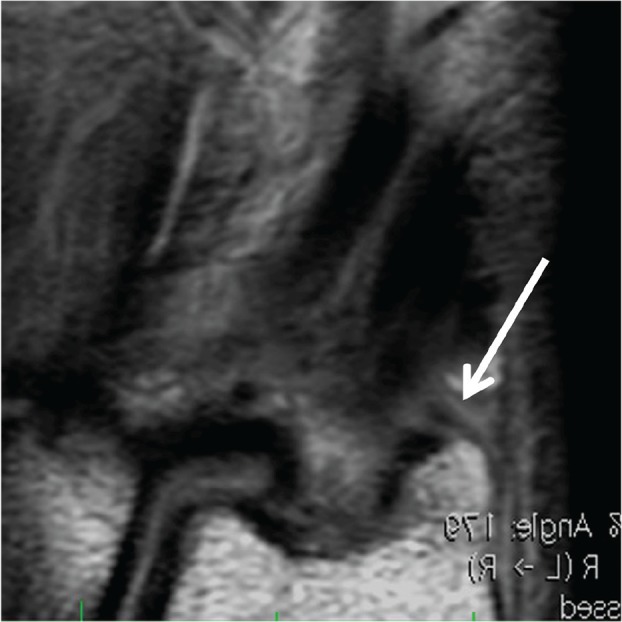
The ulnar collateral ligament (UCL) on coronal proton density (PD)-weighted image. The UCL merges from the tip of ulnar styloid process (arrow).

The UCL is a thin fibrous ligament that lies immediately superficial to the meniscus homologue.^36^ However, it is controversial whether the UCL is actually present or not. Recently, many researchers consider the “ulnar collateral ligament” of the wrist as a term describing the ulnar capsule and function rather than a distinct anatomical structure, such as the medial collateral ligament of the knee or the UCL of the elbow.^37^ Ishii used the term “ulnar capsule” which spans the border of the TFCC from the ulnotriquetral ligament to the sheath of the ECU, blending with the dorsal ulnar aspect of the ulnar styloid.^38^ In most cases, it blends seamlessly with the ECU subsheath and the meniscus homologue.^39^ This component is mainly assessed on high-resolution coronal or oblique coronal MR images (Fig. 8).

### Ulnolunate ligament, ulnotriquetral ligament

**Fig 9.**
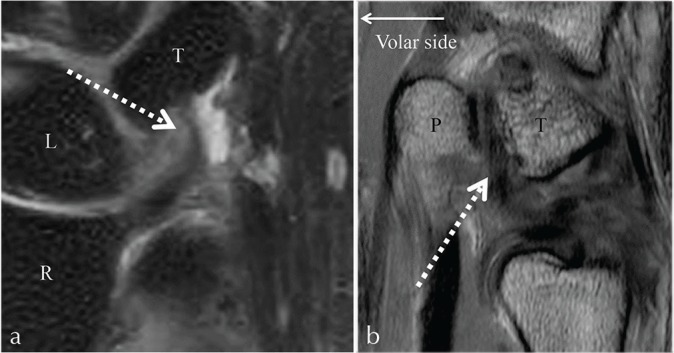
The ulnotriquetral ligament on isotropic 3-dimensional (3D) coronal fat suppressed (FS) proton density (PD)-weighted image (**a**), and on 2D sagittal PD-weighted image (**b**). The ulnotriquetral ligament shows low intensity (dotted arrow). T, triquetrum; L, lunate; R, radius; P, pisiform.

**Fig 10.**
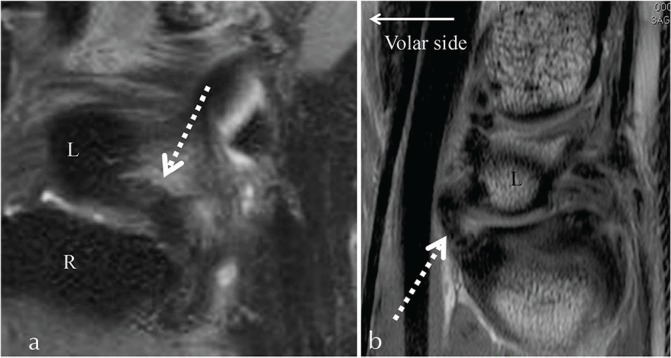
The ulnolunate ligament on isotropic 3-dimensional (3D) coronal fat suppressed (FS) proton density (PD)-weighted image (**a**), and on 2D sagittal PD-weighted image (**b**). The ulnolunate ligament shows low intensity (dotted arrows). R, radius; L, lunate.

The volar aspect of the TFCC is composed of the volar radioulnar ligament and the ulnocarpal ligaments. The ulnocarpal ligaments are anatomically divided into the ulnocapitate, ulnotriquetral, and ulnolunate ligaments. Although Palmer et al. first reported only the ulnotriquetral and ulnolunate ligaments, they have been further anatomically characterized to include the ulnocapitate ligament.^40^ Collectively, these ligaments are often titled as the ulnocarpal ligamentous complex (UCLC). The UCLC merges firmly with the palmar radioulnar ligaments, and on a coronal plane they are seen extending in a fan shape distally to insert at the palmar aspects of the triquetrum, capitate, and lunate. Macroscopically, these ligaments are often confluent and indistinguishable from each other, which resemble the glenohumeral ligaments in the shoulder. However, they are most easily assessed on high-resolution sagittal MR and coronal images (Figs. 9, 10).

## Traumatic and Degenerative Lesions of the TFCC

The injury of the TFCC is a well-recognized cause of the ulnar-sided wrist pain. The TFCC may be injured in its horizontal portion, peripheral portion, or at its attachments. In conjunction with TFCC injury, adjacent structures including cartilage or ligaments may become injured or affected.

The Palmer classification (Table 3) categorizes the TFCC lesions as traumatic (type 1) or degenerative lesions (type 2). The traumatic injuries occur far less common than degenerative lesions. The traumatic injuries of the TFCC most commonly result from a fall on a pronated outstretched upper extremity (producing an axial loading force) or a hyperpronation injury to the forearm. The traumatic tears are more common at the periphery of the TFCC, in the vascular zone. The degenerative problems of the TFCC will result from repetitive loading of the ulnar aspect of the wrist,^27^ as seen in ulnar impaction syndrome.

This classification system is frequently used by hand surgeons to determine the mechanism of injury and directing clinical management.^41^ Nonsurgical management includes activity modification, immobilization, and anti-inflammatory medications.^42,43^ Several surgical options may be considered if conservative measurements have been unsuccessful.

## Class 1 Lesions (Traumatic)

The traumatic lesions are classified according to the exact location of the injury. The surgical treatments are based upon the location of the TFCC lesion. Typically, if the lesion is within the vascular zone, the traumatic lesions are repaired. The lesions within the avascular zone are debrided.

**Fig 11.**
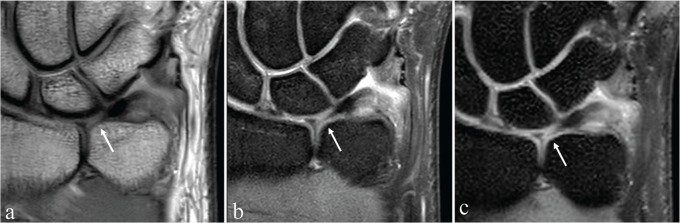
Triangular fibrocartilage complex (TFCC) central perforation (Palmer Class 1A lesion). Coronal proton density (PD)-weighted image (**a**), coronal fat suppressed (FS) PD-weighted image (**b**), and isotropic 3-dimensional (3D) coronal FS PD-weighted image (**c**) demonstrates a defect of the central disc (white arrows).

Class 1A: Class 1A lesions are traumatic tears or perforations of the horizontal portion of the TFCC (Fig. 11). These tears are dorsal–palmar slit tears that are located approximately 2–3 mm medial to the radial attachment of the TFC. Occasionally, there is a flap of cartilage that is attached only at the palmar aspect of the slit. Since the avascular articular disc has limited healing capacity, arthroscopic debridement using a motorized shaver and radiofrequency probe is the surgical treatment of choice.^44,45^

**Fig 12.**
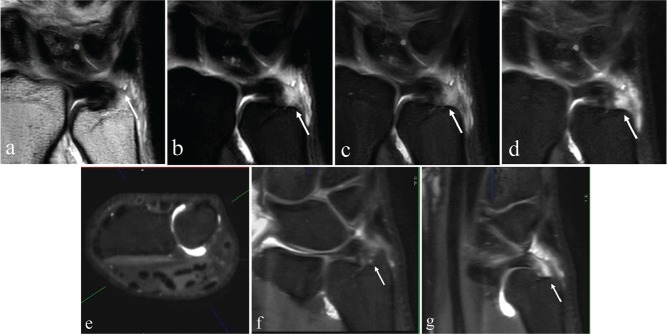
Ulnar attachment tear of the triangular fibrocartilage complex (TFCC) (Palmer Class 1B lesion). Images from an magnetic resonance arthrogram (MRA) including coronal proton density (PD)-weighted image (**a**), coronal fat suppressed (FS) T_1_-weighted image (**b**), coronal FS PD-weighted image (**c**), isotropic 3D coronal FS PD-weighted image (**d**), axial multiplanar reconstruction (MPR) (**e**), oblique coronal MPR (**f**), and oblique coronal MPR (**g**) shows traumatic avulsion of the TFCC from its insertion on the ulnar fovea (arrows except for [**e**]). Axial MPR (**e**) is used for a reference image to identify the orientation of oblique coronal MPR (**f** and **g**) at the level of the wrist joint (green line).

Class 1B: Class 1B lesions represent traumatic avulsions of the TFCC from its insertion onto the ulnar fovea and styloid process (Fig. 12). This can be associated with a fracture at the base of the ulnar styloid. As the TFCC represents the major stabilizer of the distal radioulnar joint, these lesions can often be associated with distal radioulnar joint (DRUJ) instability.^27^ It is important to test for instability of the ulnar head because this can lead to progressive osteoarthritis of the DRUJ.

The current consensus is that surgical repair should be attempted either arthroscopically or using an open technique.^46^ There are a variety of open surgical techniques that are utilized. In open techniques, the ulnar peripheral margin of the TFCC is reattached by drill holes placed through the insertion of the TFCC into the fovea of the ulnar styloid.^46^ There has been a trend towards minimally invasive surgery with arthroscopic repair.^47^ These arthroscopic techniques are similar to those for meniscal repair^48–53^ as the peripheral TFCC behaves much like the meniscus in terms of vascularity and healing potential.^47^ The arthroscopic techniques can minimize some of the complications associated with the open techniques which includes extra incisions, prominent subcutaneous suture knots, skin problems secondary to a button tied to the skin, and septic arthritis from prominent buttons and sutures.^47^ Estrella et al. retrospectively reviewed the functional outcome of arthroscopically repaired TFCC injuries.^54^ Seventy-four percent of patients achieved good to excellent results with a reduction in pain, improved grip strength, and improvement in daily activities. Seven out of nine second- look arthroscopies showed healing.

**Fig 13.**
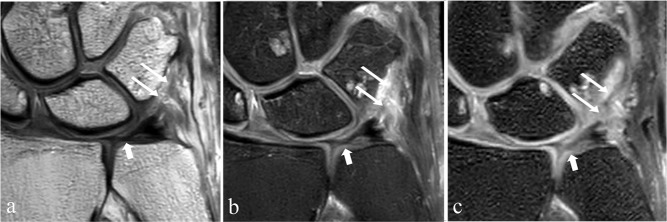
The traumatic tear of the triangular fibrocartilage complex (TFCC), specifically involving the ulnotriquetral ligaments (Palmer Class 1C) and degenerative thinning of the central disc without perforation (Palmer Class 2A). Coronal proton density (PD)-weighted image (**a**), coronal fat suppressed (FS) PD-weighted image (**b**), and isotropic 3-dimensional (3D) coronal FS PD-weighted image (**c**) demonstrates the ulnotriquetral ligament tear (thin white arrows) and thinning of the central disc (thick white arrows).

Class 1C: Class 1C lesions represent traumatic tears of the TFCC in its periphery, specifically involving the ulnolunate or ulnotriquetral ligaments (Fig. 13). This type of injury can result in ulnocarpal instability with palmar translocation of the ulnar carpus in relationship to the radius and/or ulnar head. For small tears, arthroscopic repair is recommended; for large tears, an open procedure is used.^55^ The repair is augmented using a portion of the flexor carpi ulnaris tendon.

Class 1D: Class 1D lesions are traumatic avulsions of the TFCC from its radial attachment at the distal aspect of the sigmoid notch. These injuries can be accompanied by distal radial fractures. The earlier studies on debridement reported ∼80% good results.^56,57^ Bednar et al. were among the first to describe the arthroscopic repair of radial TFCC lesions,^58^ subsequently, many surgical repairs have been reported^50,51,58–60^ with the comparison between repair and debridement showing similar outcomes.^61^ In approximately half of the cases of TFCC tear, the spontaneous repair with ulnar shortening was found. There have also been spontaneous repairs of tears to the disc proper.^62^

## Class 2 Lesions (Degenerative)

The degenerative lesions are classified according to the extent and location of the degenerative changes.

Class 2A: Class 2A lesions represent degenerative wear or thinning of the central disc of the TFCC, without perforation (Fig. 13). The degenerative changes of the TFCC will manifest as increased internal signal intensity of the fibrocartilaginous disc in a globular or curvilinear spatial pattern without articular surface extension.

Class 2B: Class 2B lesions represent the degenerative wear of the central disc of the TFCC with additional chondromalacia of the lunate and/or ulna.

If conservative therapies fail for Class 2A and 2B lesions, ulnocarpal unloading may be performed with an ulnar shortening procedure. This can be accomplished by an extra-articular ulnar-shortening osteotomy or an arthroscopic intra-articular wafer resection of the ulna.^63^

Class 2C: Class 2C lesions demonstrate the progression of degenerative changes with perforation of the central disc of the TFCC. These perforations are usually located within the central, avascular portion of the TFCC, and tend to have a more oval configuration.^27,41^ If conservative therapies fail in 2C lesions, there are three surgical options including: arthroscopic TFCC debridement, the ulnar wafer procedure, or ulnar shortening.

**Fig 14.**
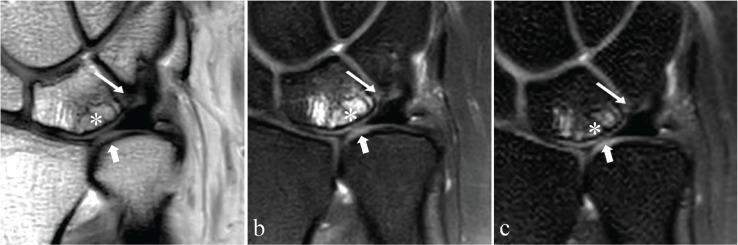
Degeneration with perforation of the central disc of the triangular fibrocartilage complex (TFCC), chondromalacia of the lunate and/or ulna, and lunotriquetral ligament (LTL) disruption (Palmer Class 2D). Coronal proton density (PD)-weighted image (**a**), fat suppressed (FS) PD-weighted image (**b**), and isotropic 3-dimensional (3D) FS PD-weighted image (**c**) shows the degeneration and perforation of the central disc (thick arrows), chondromalacia of the lunate (*) and LTL disruption (thin arrows).

**Fig 15.**
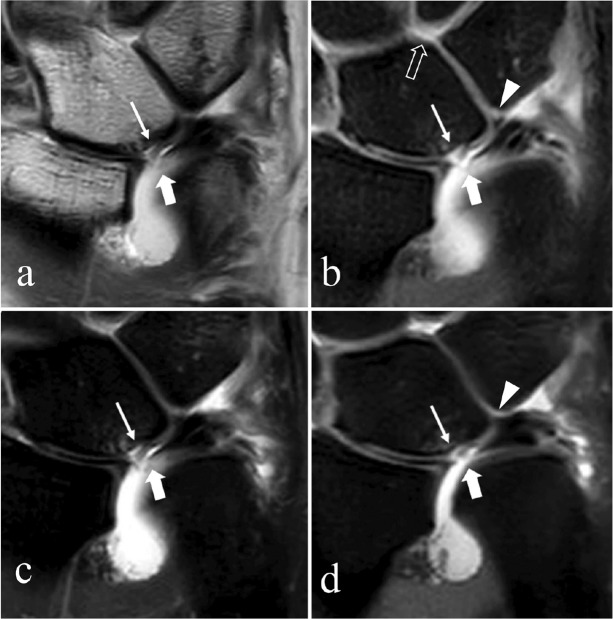
Degeneration with perforation of the central disc of the triangular fibrocartilage complex (TFCC), chondromalacia of the lunate and/or ulna, and lunotriquetral ligament (LTL) perforation (Palmer Class 2D). Magnetic resonance arthrogram (MRA) coronal proton density (PD)-weighted image (**a**), coronal fat suppressed (FS) T_1_-weighted image (**b**), coronal FS PD weight image (**c**), and isotropic 3-dimensional (3D) coronal FS PD-weighted image (**d**) shows the degeneration and perforation of the central disc (thick arrows) and chondromalacia of the lunate with cartilage flap (thin arrows). A small and irregularly shaped LTL with oblique linear high signal (contrast) though its membranous portion (arrowheads) is visualized in (**b** and **d**), and intra-articular contrast extends into the midcarpal joint (open arrow in [**b**]), suggesting a LTL perforation.

Class 2D: Class 2D lesions represent even further progression of the degenerative changes with perforation of the central disc of the TFCC, chondromalacia of the lunate and/or ulna, and lunotriquetral ligament (LTL) disruption (Figs. 14, 15). The surgical options include debridement of the TFCC and LTL, chondroplasty, possible arthroscopic reduction, and internal fixation of the lunotriquetral interval if unstable and/or ulnar shortening.^43^ Ulnar shortening is utilized in these lesions, as it stabilizes the lunotriquetral joint by increasing tension on the LTL. An isolated wafer procedure is not utilized in these lesions, as it unloads the ulnocarpal joint, but does not stabilize the lunotriquetral joint.

Class 2E lesions: Class 2E lesions represent the final stages of the ulnar impaction syndrome with large central disc TFCC perforation, chondromalacia, and LTL disruption. These findings are often accompanied by ulnocarpal and distal radioulnar degenerative arthritis. The treatment options for this end stage of ulnar impaction include hemiresection arthroplasty salvage procedures, such as Darrach or Sauve–Kapandji procedures.

## Figures and Tables

**Table 1.** Routine magnetic resonance (MR) parameters for 3Tesla (T) imaging of the wrist

Sequence	Cor 3D PD FS	Cor PD	Cor PD FS	Ax PD	Ax PD FS / Ax T_2_ FS	Sag PD	Sag PD FS
Pixel size (mm × mm)	0.35 × 0.35	0.27 × 0.35	0.30 × 0.32	0.4 × 0.4	0.32 × 0.44	0.40 × 0.51	0.23 × 0.32
Slice thickness (mm)	0.35	2	2	2	2	3	3
Slice gap (mm)	0	0.2	0.2	0.2	0.2	0.3	0.3
Number of slices	151	22	22	30	30	22	20–30
FOV (mm)	70	90	90	80	80	70–80	70–80
TE (ms)	29–37	30	27	30	30/70	30	27
TR (ms)	1250–1400	3500	2500–3500	2500	3000/3200	3000	2500–3500
BW (Hz/pixel)	362	194	222	250	153/218	354	175
Echo train length	70–88	13	13	9	11	12	13
NEX	2	1	3	2	2	1	1
Acquisition time (min)	∼5	∼2.5	∼4	∼3	∼2.5/3	∼2.8	∼3.5

Ax, axial; Cor, coronal; Sag, sagittal; 3D, 3-dimensional; PD, proton-density; FS, fat-suppression; FOV, field-of-view; TE, echo time; TR, repetition time; BW, bandwidth; NEX, number of excitations.

**Table 2.** Additional sequences for magnetic resonance arthrogram (MRA) of the wrist

Sequence	Ax T_1_ FS	Cor T_1_ FS	Sag T_1_ FS
Pixel size (mm × mm)	0.4 × 0.4	0.4 × 0.4	0.5 × 0.5
Slice thickness (mm)	2	2	2
Slice gap (mm)	0.2	0.2	0.2
Number of slices	30	20	27
FOV (mm)	70–80	80	70
TE (ms)	23	23	23
TR (ms)	631	630	570–650
BW (Hz/pixel)	353	354	444
Echo train length	3	3	3
NEX	2	2	2
Acquisition time (min)	∼4.2	∼3	∼3

Ax, axial; Cor, coronal; Sag, sagittal; FS, fat-suppression; FOV, field-of-view; TE, echo time; TR, repetition time; BW, bandwidth; NEX, number of excitations.

**Table 3.** Palmer classification categorizing triangular fibrocartilage complex (TFCC) lesions as traumatic (type 1) or degenerative (type 2)

Type 1: Traumatic injury	Type 2: Degenerative injury
1A: Central perforation	2A: TFCC wear
1B: Ulnar avulsion with or without distal ulnar fracture	2B: TFCC wear with lunate and/or ulnar chondromalacia
1C: Distal avulsion	2C: TFCC perforation with lunate and/or ulnar chondromalacia
1D: Radial avulsion with or without sigmoid notch fracture	2D: TFCC perforation with lunate and/or ulnar chondromalacia and lunotriquetral ligament perforation
	2E: TFCC perforation with lunate and/or ulnar chondromalacia, lunotriquetral ligament perforation, and ulnocarpal arthritis
